# The Role of Antibiotic Resistant *A. baumannii* in the Pathogenesis of Urinary Tract Infection and the Potential of Its Treatment with the Use of Bacteriophage Therapy

**DOI:** 10.3390/antibiotics10030281

**Published:** 2021-03-09

**Authors:** Natalia Bagińska, Martyna Cieślik, Andrzej Górski, Ewa Jończyk-Matysiak

**Affiliations:** 1 Bacteriophage Laboratory, Hirszfeld Institute of Immunology and Experimental Therapy, Polish Academy of Sciences, 53-114 Wrocław, Poland; natalia.baginska@hirszfeld.pl (N.B.); martyna.cieslik@hirszfeld.pl (M.C.); agorski@ikp.pl (A.G.); 2 Phage Therapy Unit, Hirszfeld Institute of Immunology and Experimental Therapy, Polish Academy of Sciences, 53-114 Wrocław, Poland; 3 Infant Jesus Hospital, The Medical University of Warsaw, 02-006 Warsaw, Poland

**Keywords:** bacteriophages, multidrug resistance (MDR), urinary tract infection (UTI), uropatogenic bacteria, critical priority group

## Abstract

*Acinetobacter baumannii* are bacteria that belong to the critical priority group due to their carbapenems and third generation cephalosporins resistance, which are last-chance antibiotics. The growing multi-drug resistance and the ability of these bacteria to form biofilms makes it difficult to treat infections caused by this species, which often affects people with immunodeficiency or intensive care unit patients. In addition, most of the infections are associated with catheterization of patients. These bacteria are causative agents, inter alia, of urinary tract infections (UTI) which can cause serious medical and social problems, because of treatment difficulties as well as the possibility of recurrence and thus severely decrease patients’ quality of life. Therefore, a promising alternative to standard antibiotic therapy can be bacteriophage therapy, which will generate lower costs and will be safer for the treated patients and has real potential to be much more effective. The aim of the review is to outline the important role of drug-resistant *A. baumannii* in the pathogenesis of UTI and highlight the potential for fighting these infections with bacteriophage therapy. Further studies on the use of bacteriophages in the treatment of UTIs in animal models may lead to the use of bacteriophage therapy in human urinary tract infections caused by *A. baumannii* in the future.

## 1. Introduction

**Figure 1:**
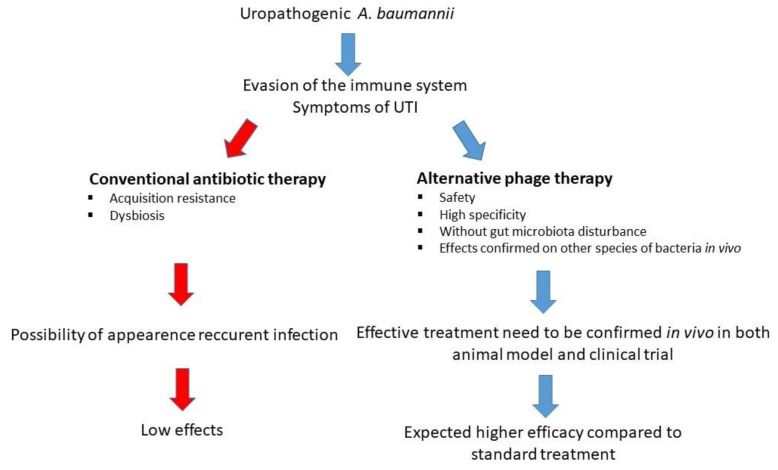
Comparison of antibiotic therapy and the potential new alternative phage therapy in combating uropathogenic strains of *A. baumannii*.

**Figure 2:**
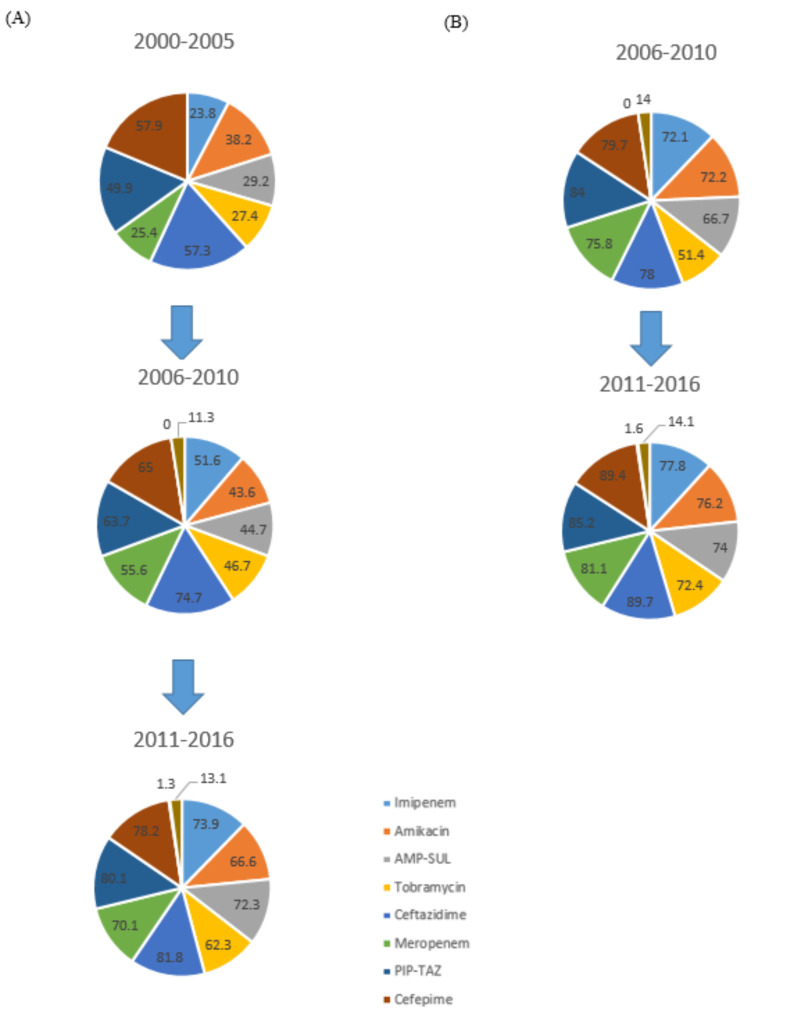
Resistance of *A. baumannii* strains in the world to available antibiotics in countries belonging to the Organization for Economic Co-operation and Development (OECD) (**A**) and non‑OECD (**B**). PIP-TAZ piperacillin-tazobactam, AMP-SUL ampicillin-sulbactam [11].

Each year, approximately 150 million people worldwide suffer from UTI [1]. They are caused by uropathogenic bacteria, such as *Escherichia coli*, *Klebsiella, Staphylococcus*, *Enterococcus* [2], and multi-drug resistant (MDR) *Acinetobacter baumannii* [3]. In addition to bacteria, urogenital infections can be caused by the sub-Saharan Africa endemic parasite *Schistosoma haematobium* [4] or by *Candida* species. Fungal infections of the urinary tract caused by *Candida* affect people with weakened immunity or those who are hospitalized [5]. Infection of the urinary tract causes many difficulties during treatment due to the increasing antibiotic resistance among bacterial strains [6]. Approximately 60% of women will experience a UTI at least once in their lifetime, and 20–30% of them will have had a recurrence within 6 months [6,7,8]. The costs incurred in the fight against UTI are related to the treatment, but also to the loss of productivity related to absence from work [6]. The common method of fighting UTI is antibiotic therapy. A huge problem arose when the widespread use of antibiotics resulted in the appearance of antibiotic-resistant bacterial strains all over the world. Therefore, other possibilities of treatments for UTI should be urgently considered [9]. One of the promising treatment methods is bacteriophage therapy (Figure 1), which can prove to be an effective method of combating this serious medical and social problem [10]. The increase in resistance among *A. baumannii* to currently used drugs is shown in Figure 2.

*A. baumannii* most often causes hospital bacteremia and lung infections. The presence of an endotracheal tube creates an ideal condition for the formation of bacterial biofilm, which results in infection [12]. Bloodstream infections caused by *A. baumannii* are related to the presence of the central venous catheter or as a consequence of extensive pneumonia. Additionally, *A. baumannii* causes infections of the urinary tract, usually associated with the presence of percutaneous nephrostomy tubes or urinary catheters. These pathogenic strains are also responsible for meningitis, osteomyelitis, endocarditis and wound infections. These infections occur mainly after an injury or surgery [13,14,15,16,17]. *A. baumannii* isolates are characterized by multi-drug resistance, therefore the treatment of infections caused by these bacteria is quite limited. Standard treatment of susceptible strains is based on the use of beta lactam antibiotics. In more difficult cases, carbapenems are applied [18,19,20]. The growing resistance among *A. baumannii* to currently used therapeutics has forced a search for a new effective, as well as safe method of fighting these infections, such as bacteriophage therapy [21]. Bacteriophages can be isolated from various environments [22,23,24]. Those specific to *A. baumannii* are most often isolated from water samples, especially from hospital sewage and almost every part of the human body [25,26,27,28,29]. When examining human samples, bacteriophages were detected in 11% of blood cultures, 14% of cerebrospinal fluid, 23% of urine samples, 28% of serum and 45% of ascitic fluid [30]. So far, rodent studies have shown that bacteriophages specific to a particular strain of *A. baumannii* can be helpful in combating infection (intraperitoneal sepsis, wound or lung infection) caused by this pathogen [31,32,33,34].

### 1.1. Global Problem of the Urinary Tract Infections Caused by A. baumannii

In recent years, all over the world, among nosocomial pathogens, an increasing number of infections have been caused by Gram-negative bacteria. Additionally, many of them are MDR strains [35]. MDR *A. baumannii*, formerly considered commensals, nowadays are associated with many dangerous hospital infections [36]. These strains can easily be isolated from intubated patients in the intensive care unit (ICU) [37]. Many of these strains are MDR; Lob et al. (2016) in their work described research on *A. baumannii* strains isolated from patients in 48 countries (453 hospitals) around the world under the Study for Monitoring Antimicrobial Resistance Trends (SMART) [38]. In this study from 2011 and 2014, 2337 *A. baumannii* isolates were collected. These strains were isolated from patients with intra-abdominal infections and UTIs, and for a group of 1011 isolates (2013–2014), sensitivity and multidrug resistance (resistant to at least three classes of drugs) were determined. Of these, 721 were from patients with intra-abdominal infections and 276 isolates were from patients with UTI. A total of 307 isolates came from intensive care units (ICU), and 615 from other hospital units.

Drug resistance was tested for the isolates of *A. baumannii*. Among the strains causing UTI in the Middle East, over 90% of the strains were MDR, similar to Europe 90% of *A. baumannii* strains were MDR. In Latin America, Africa, Asia and North America, approximately 85%, 80%, 70% and 50% of isolates were MDR, respectively. It is worth noting that in these regions of the world (except North America) over 90% of isolated *A. baumannii* strains in intensive care units were MDR. For North America, more than 60% of *A. baumannii* isolates were MDR, and this phenomenon remains unclear [38].

### 1.2. Acinetobacter baumannii as an Uropathogenic Species

Jiménez-Guerra et al. (2018) described the evolution of *A. baumannii* resistance in UTIs [39]. Among the isolated strains of *A. baumannii*, the highest percentage value of resistance was demonstrated for fosfomycin, aztreonam, ciprofloxacin, ceftazidime and cefepime. In vitro studies showed that colistin turned out to be the most effective antibiotic against these bacteria. Additionally, in the years 2013–2016 an increase in the resistance of *A. baumannii* isolates to imipenem, piperacillin-tazobactam and meropenem was noted. In addition to antibiotic resistance, *A. baumannii* may be resistant to bacteriophages. However, bacteriophage resistance may become a desirable trait. In vitro studies on two strains of *A. baumannii* (AB900 and A9844) showed that with the acquisition of resistance to specific bacteriophages (ΦFG02 and ΦCO01, respectively) the bacteria lost their capsule on the bacterial surface. The loss of capsule was due to a defect in the overall production of capsule polysaccharides in bacteriophage-resistant bacteria. This defect contributed to a 2 × decrease in the minimum inhibitory concentration (MIC) for ciprofloxacin and as much as a 16 × decrease in the MIC for ceftazidime for the bacteriophage resistant AB900 strain compared to the wild strain. For bacteriophage resistant strain A9844, a 2 × decrease in MIC was observed for minocycline, meropenem, cefepime and ampicillin with sulbactam compared to the wild strain. Additionally, resistance to bacteriophages of *A. baumannii* strains contributed to their sensitivity to the action of human complement [40].

Natural physiological mechanisms, such as the physicochemical properties of urine (e.g., the correct pH), urination, or the structure of the urethra, prevent bacteria from entering the sterile parts of the urinary tract. Some virulent organisms, known as uropathogens, have the ability to overcome natural protective barriers. This may be favored by the presence of adhesive fimbriae, exemplified by the synergistically acting type P and type 1 fimbriae in uropathogenic *E. coli*, which significantly promotes colonization of renal tubules [41]. Another important virulence factor—proteases, have also been described in UTI-causing *Acinetobacter* strains [42,43]. Important enzymes encoded by bacteria and conducive to the development of infections, including UTIs, are haemolysins and aerobactins [44], which are involved in the acquisition of iron necessary for bacterial growth. 

Another group of enzymes are phospholipases, which contribute to an increase in bacterial virulence by degrading the phospholipids of human cell membranes [45]. Although *Acinetobacter* is considered a non-motile bacterium, it has been shown to be capable of twitching motility, which may contribute to it spreading more easily [46]. *A. baumannii* is also characterized by an escape from the human immune system by removing zinc ions, thanks to which bacterial growth is not inhibited by one of the proteins of the immune system—calprotectin. High resistance to dryness and the ability to pump out antiseptics significantly contribute to the difficulties in fighting this pathogen [45].

Di Venanzio et al. (2019) conducted an analysis of *Acinetobacter* isolates identified in the BJC Healthcare System (BJC) in the period from January 2007 to August 2017 [43]. Among 2309 cases of *Acinetobacter calcoaceticus*-*baumannii* complex (Acbc), 22.2% Acbc was isolated from urinary sources; other sources of Acbc isolation were respirators (33.9%), soft tissue/musculoskeletal (31.9%) and endovascular (10.4%). The authors made an even broader analysis, reviewing clinical trials since 1995, which took into account the anatomical location of Acbc or *A. baumannii* isolates. An analysis of over 19,000 cases showed that the majority of isolates came from the respiratory tract (39.5%) and soft tissue/musculoskeletal (22.7%). The percentage share of uropathogenic isolates ranged from 6.1% to 29.3%, and the average was 17.1% of all isolates (3410 from 19,957). From the above analysis it can be concluded that *A. baumannii* strains constitute a significant percentage of pathogens infecting the urinary tract (Table 1).

The analysis shows that on average, 17.1% of *A. baumannii* strains are isolated from urinary sources, but only 2% of UTIs are caused by these bacteria. However, *A. baumannii* is the main pathogen causing UTIs associated with the use of catheters in intensive care units [59,60]. More than half of *A. baumannii* strains isolated from urine come from catheterized patients. Moreover, other infections caused by *A. baumannii* are often associated with the use of medical equipment, such as central venous lines or endotracheal [61]. Di Venanzio et al. (2019), to investigate the uropathogenesis of *A. baumannii,* used the catheter-associated UTI mouse model [43]. This model is frequently used to study the uropathogenic methicillin-resistant *Staphylococcus aureus* (MRSA), Group B *Streptococcus, Escherichia coli* and *Enterococcus faecalis* [62,63,64]. Two strains of *A. baumannii* isolated from urine were used to test the ability to colonize the kidneys and bladder of mice: UPAB1 and ATCC19606. The isolated MDR UPAB1 strain came from a patient with uncomplicated UTI; the ATCC19606 strain is widely used to study the virulence of *A. baumannii* in murine models of sepsis and pneumonia [43,65]. The bacteria were administered through an implant (transurethrally by a small piece of silicone tube) placed in the animal’s urethra [43]. The results of the experiment show that infection with the UPAB1 strain caused a 5 log increase in bacterial count on the implants and in the bladders of mice 24 h after infection. A fluorescence microscope confirmed the presence of bacteria on the silicone implants and luminal urothelial surface, whereas the ATCC19606 strain was almost completely removed from the body of the mice 24 h after infection. This study showed that UPAB1 infects the urinary tract like other uropathogenic bacteria.

Sequencing of the UPAB1 genome indicated that this strain possesses the pAB5 plasmid, the presence of which affected uropathogenesis and was associated with a higher bacterial titer on the implant and urinary bladder of mice in the catheter-associated UTI (compared to the control without the plasmid). Completely different results were obtained for lung infection in mice caused by the UPAB1 strain. In the case of the wild-type strain (with the pAB5 plasmid), 36 h after intranasal administration, the bacterial titer was 1 to 4 logs lower in livers, lungs, kidneys, spleens and hearts compared to the infection with the strain without the plasmid. In addition, no deaths were reported among mice infected with the wild-type bacterial strain where infection with the strain without plasmid resulted in 40% mortality in the animals. The results suggest that *A. baumannii* strains show different survival rates depending on the anatomical location of the host, and additional studies are needed to thoroughly investigate their pathobiology [43].

In the United States, approximately 100,000 cases of catheter-UTIs are reported each year [66]. This may be due to the formation of a bacterial biofilm on the surface of the catheter [67]. Even more than 75% of *A. baumannii* strains are capable of producing biofilm, therefore the bacteria may contribute significantly to UTIs [68,69,70]. Braun and Vidotto (2004) isolated 13 strains of *A. baumannii* derived from urinary sources from patients aged 18 to 88 years. Six strains have been isolated from hospital patients and seven from outpatients. The susceptibility of these strains to selected antibiotics was tested, three strains from non-hospitalized patients were sensitive to the action of most of the antibiotics used. Interestingly, the remaining strains turned out to be resistant to amikacin, ceftriaxone, ciprofloxacin and trimethoprim-sulfamethoxazole. [71]. Pour et al. (2011) also described uropathogenic strains of *A. baumannii*. Researchers isolated 47 strains of *A. baumannii* and three strains of *A. lwoffii* from urinary tract and urinary catheter samples. The ability of the tested strains to produce biofilm was also assessed, and it turned out that biofilm forms better on polypropylene than on glass surfaces. Additionally, shaking promoted the formation of a bacterial biofilm. What is more, resistance to 27 different antibiotics was tested for six biofilm-forming strains of *A. baumannii*. These isolates were resistant to 97% of aminoglycosides, 94.4% of the cephalosporin group, 83.3% of β-lactams, 75% of quinolones, 66.6% of tetracycline and oxytetracycline, 33.3% of imipenem and 50% of the other antibiotics tested. Interestingly, all isolates were sensitive to colistin [72].

*A. baumannii* infections do not only affect humans, they also cause infections in animals. Kuzi et al. (2016) described UTIs in hospitalized dogs and cats caused by *Acinetobacter calcoaceticus*-*baumannii* complex (Acbc) [73]. A total of 19 dogs and four cats were hospitalized due to the primary disease. Twenty-two animals were treated in the intensive care unit and five of them were transferred to the general hospitalization ward after 1–3 days. The animals were hospitalized for 2 to 15 days. There were clinical signs among the animals (including: weakness 52%, fever 39%, urine turbidity 30%) indicative of a healthcare-associated secondary Acbc infection (pneumonia, UTI, sepsis, cellulitis and septic arthritis). In total, 10 hospitalized animals (two cats and eight dogs) were diagnosed with UTI and the Acbc was isolated from the urine in two cats and seven dogs. All animals diagnosed with secondary Acbc urinary tract infection were previously catheterized. Antibiotics were given to all animals diagnosed with Acbc infection. As a result of infection, all dogs with the infection survived and two cats died [73].

Bacterial strains isolated from animals are also characterized by a significant increase in MDR [74]. The Acbc strains were isolated from cats and dogs from urine, blood, bronchoalveolar lavage fluid, kidneys, lungs, heart, and liver, as well as fluid aspirated from subcutaneous tissues with cellulitis. For all isolated Acbc strains, the antibiotic resistance profile was investigated. All of them were resistant to most of the tested antibiotics commonly used in hospitals: amoxicillin-clavulanic acid, amikacin, ampicillin, fluoroquinolones, gentamycin, sulfamethoxazole/trimethoprim, polymyxin B, florfenicol, third generation cephalosporins, second generation cephalosporins, first generation cephalosporins, imipenem and ticarcillin. The isolated strains turned out to be sensitive only to polymyxin-B (96%), imipenem (74%), ticarcillin (59%) and amikacin (35%). The above study shows that veterinary MDR Acbc infections are characterized by high mortality up to 70% [73].

It is important to remember that there is a significant problem regarding the possibility of transmitting zoonotic pathogens to humans, including bacteria from the ESKAPE group, such as *A. baumannii* [75]. The role of animals, especially dogs, in helping in the treatment of hospitalized patients is clearly emphasized, and despite their many benefits, they can pose a serious threat as carriers of pathogens.

### 1.3. Bacteriophages as a Tool in the Fight against Uropathogenic Bacteria

Bacteriophages were discovered over 100 years ago, first by microbiologist Frederick Twort in 1915 [76]. Then, in 1917, Felix d’Herelle published a paper about bacteriophages, as invisible microbes that were present in the filtrate of feces of people suffering from dysentery. He showed that the titer of bacteriophages increases with the development of the disease and reaches its highest value during recovery. d’Herelle tested bacteriophage therapy on himself and his relatives, and then on patients with dysentery and cholera. It was then used to heal wounds, and the bacteriophages were tested for avian typhosis caused by *Salmonella gallinarum*. Bacteriophages have been used against *Pasteurella multocida* in the treatment of bovine hemorrhagic septicaemia. However, the first paper on bacteriophage therapy appeared in 1921, published by Bruynoghe and Maisin [76]. Due to the discovery of antibiotics, the use of bacteriophages has been limited to Poland and Georgia [77]. Nowadays however, because of problems with antibiotic inefficiency, this method of treating bacterial infections has gained renewed interest.

Bacteriophage therapy, unlike antibiotics, does not damage the natural microbiota of both the human and animal body. Because bacteriophages are able to be amplified only with the presence of susceptible bacteria causing lysis of their cells, they are named self-limiting “drugs”. What is more, bacteriophages may also be active against antibiotic-resistant strains of bacteria. Therefore, in people with bacterial infections, where antibiotics failed, it is possible to use lytic bacteriophages [10,28] that also proved to be safe and well-tolerated by immunocompromised patients as well as people allergic to antibiotics. This is likely due to their structure, which is composed of proteins and nucleic acids only and therefore are considered as non-toxic [78]. In contrast to antibiotics, bacterial viruses that are most abundant in the environment can thus be sought and isolated from places where bacteria are present [79,80,81]. Bacteriophages can be found in almost any environment, and thus the costs associated with the production of bacteriophage preparations are lower than in the case of antibiotics [82]. Interestingly, a particularly desirable feature of bacteriophages is their ability to combat bacterial biofilm, the structure of which is difficult to penetrate and destroy by antibiotics. In addition, the potential of bacteriophage therapy efficacy against pathogenic microorganisms has been confirmed by different studies on animal models [83,84,85,86]. Due to the features mentioned above, bacteriophage therapy has become a promising and effective alternative to standard therapy for the treatment of UTIs also caused by MDR bacteria.

In the case of UTI treatment, natural bacteriophage cocktails, bacteriophage lytic enzymes or proteins (natural or engineered form), genetically engineered bacteriophages, as well as bacteriophages in combination with antibiotics may be used. Thus, bacteriophages can be detected in the urine, and despite its alkaline pH, bacteriophages can remain active in the urine [22,87]. For example, UTIs by *E. coli* resulted in the appearance of bacteriophages specific for these bacteria in the urine [88,89]. Active bacteriophage particles specific to *E. coli* were detected in the urine after intravenous injection of purified preparation into the bloodstream [90]. Interestingly, specific bacteriophages in urine were also found in 30% of patients after oral administration of bacteriophage preparation [91].

Nowadays, bacteria are acquiring new and more sophisticated resistance mechanisms to the available antibiotics. Lytic bacteriophages are capable of destroying bacteria at the end of the infection cycle, and antibiotic resistance in the case of bacterial strains does not preclude effective bacteriophage therapy [78]. Therefore, bacteriophage therapy serves as a great tool to fight MDR bacterial infections [92], especially UTI caused by MDR *A. baumannii*.

UTIs are a serious social and economic problem. In the United States, as many as seven million medical visits and more than 100 thousand hospital admissions a year are caused by UTIs. A total of 15% of prescribed antibiotics are used to treat UTIs, at a cost of 1.6 billion dollars a year [93]. Pyelonephritis is a common case of patient UTIs and is associated with the patient’s catheterization [94]. Another problem in the fight against UTI is the widespread use of antibiotics and the increasing phenomenon of resistance acquisition among uropathogenic strains [95,96]. Bacteriophage therapy is recognized as safe and effective in the treatment of UTI, as confirmed by clinical trials for the treatment of UTI and diarrhea in children caused by enteropathogenic *E. coli*, uropathogenic *E. coli,* enterotoxigenic *E. coli*, and *P. aeruginosa* [97,98,99,100]. Therefore, bacteriophages may be a real alternative for treating UTI. Due to the bacteriophage preparations, it is possible to combat specific bacterial hosts. Compared to bacterial resistance to antibiotics, bacteriophage resistance is slight, but present among some bacterial strains [101]. Nevertheless, there are many readily available sources of searching for new bacteriophages [102,103], and the process leading to the discovery of a new bacteriophage is relatively inexpensive and fast, therefore it is quite easy to match specific bacteriophages to specific bacterial strains. The use of a bacteriophage cocktail instead of monotherapy reduces the likelihood of the emergence of bacteriophage resistance. Each of the bacteriophages can be attached to a different receptor in a bacterial cell, and different bacteriophages can also act synergistically, so using a cocktail of several bacteriophages increases the probability of therapeutic effectiveness [104,105]. 

Using an intravenous catheter, Nishikawa et al. (2008) transurethrally injected an uropathogenic *E. coli* strain ECU5 (5·10^9^ CFU/mL) into the BALB/c mouse bladder [106]. Next, the purified T4 at multiplicities of infection (MOIs): 0.01–60 or KEP10: MOI = 60 bacteriophages were administered into the peritoneal cavity. For T4 bacteriophage at MOI 0.01, 0.1, 0.5, 1 and 60 survival rate after 3 days was 40%, 60%, 40%, 80% and 100% respectively. The administration of the KEP10 at MOI = 60 rescued 90% of infected animals compared to the untreated control where 100% of the mice died [106].

Subsequently, UTI bacteriophage therapy was applied in a mouse model through the transurethral application of *Cronobacter turicensis* (10^11^ CFU/mL). At the same time, specific bacteriophages were given intraperitoneal at a titer of 10^11^ PFU/mL. After 24 h, the bacterial count in the kidneys in mice treated with bacteriophage therapy reduced the bacterial count by 70% [107].

Sybesma et al. (2016) described the effect of commercial bacteriophage cocktails on uropathogenic clinical strains of *K. pneumoniae* and *E. coli* [108] in vitro. The results of a study of 41 clinical *E. coli* strains showed that the lytic activity of the Enko, Ses, Intesti, Pyo (before adaptation) and Pyo (after adaptation) bacteriophage cocktails was 92.7%, 90.2%, 82.9%, 65.9% and 92.7%, respectively. For nine clinical *K. pneumoniae* strains, the lytic activity of 10 bacteriophages from the Eliava collection ranged from 0% (vB_KlpR1, vB_KloxR2, vB_KlpR5 and vB_KlpR7 bacteriophage), 11% (vB_KlpR6 bacteriophage), 22% (vB_KlpR3, vB_KlpR4 and vB_KlpR8 bacteriophage), 56% (v_BRKpM9 bacteriophage) up to even 100% (v_BRKpS10 bacteriophage).

Many of the uropathogenic bacterial strains have the ability to produce biofilm, especially in urinary catheters, which hinders the therapeutic effects of antibiotics [67,109,110]. The antimicrobial activity of bacteriophages against biofilm-producing *A. baumannii* strains was demonstrated in vitro in the study conducted by Vukotic et al. [111]. Two bacteriophages, isolated from Belgrade wastewater samples, were tested against 103 *A. baumannii* isolates, resulting in a higher host range for the ISTD bacteriophage compared to NOVI bacteriophage (36% and 22%, respectively). Moreover, a reduction in bacterial cell count in the biofilm formed on porous glass beads was observed 6 h after ISTD bacteriophage application. However, after 24 h of observation, the number of bacterial cells did not differ between the biofilm on which the bacteriophages were applied and the control biofilm.

The action of bacteriophages combined with antibiotics on the reduction in biofilm produced by uropathogenic strains of *A. baumannii* was tested in vitro [112]. Among 25 clinical uropathogenic strains of *A. baumannii*, one was selected AB20, which was characterized by a strong biofilm formation capacity, sensitivity to the bacteriophage used (Aba-1–Aba-6) and resistance to antibiotics (ciprofloxacin, meropenem, levofloxacin, trimethoprim/sulfamethoxazole, netilmicin gentamicin, and imipenem). The bacterial biofilm produced after 24 h, immersed in human urine, was treated with 1/2 MIC and 1/4 MIC of antibiotics: ciprofloxacin (MIC = 8), levofloxacin (MIC = 16), trimethoprim/sulfamethoxazole (MIC = 64), amikacin (MIC = 32), tobramycin (MIC = 2), gentamicin (MIC = 64), colistin (MIC = 1), imipenem (MIC = 16), meropenem (MIC = 32). Then, a bacteriophage cocktail (Aba-1, Aba-2, Aba-3, Aba-4, and Aba-6) with a titer of 1·10^7^ PFU/mL was added to each sample with an antibiotic. After 6 h of incubation, the highest level of biofilm biomass reduction was recorded for bacteriophages combined with trimethoprim/sulfamethoxazole 98.6% and 94.3% for ½ and ¼ MIC, respectively. The use of ciprofloxacin in combination with the bacteriophage cocktail resulted in a reduction of 93.3% and 87% for ½ and ¼ MIC, respectively. For the remaining antibiotics, the results were similar, only amikacin and levofloxacin did not act synergistically with bacteriophages. Additionally, the weakest result was recorded for the combination of the bacteriophage cocktail with colistin, where increased biofilm formation was noted. The bacteriophage cocktail alone reduced the biofilm biomass in 77%. These studies indicate that bacteriophage therapy in the future may be a useful tool in the fight against UTIs in humans. So far, no studies have been conducted on the use of bacteriophage therapy in the treatment of UTIs caused by *A. baumannii* in an animal model. However, other work on treating UTIs caused by uropathogenic strains (*E. coli*, *P. aeruginosa*, *Cronobacter* spp.) has shown positive results [97,98,99,100,107]. Due to the differences between the human body and the organisms of various animals, the results obtained in vivo studies with the use of animal models cannot be fully related to the human body, but they significantly help, e.g., in choosing the most advantageous route of bacteriophage administration or assessing the appropriate therapeutic dose [113].

The possibility of using bacteriophage-derived enzymes as effective tools to combat UTI is also emphasized [114]. Despite the satisfactory results of the action of bacteriophage lysins mainly on Gram-positive bacteria, promising studies of these enzymes in *A. baumannii* sepsis in a mouse model have been conducted [115].

Bacteriophages are capable of fighting bacterial biofilm that can form on the catheters. Additionally, bacteriophages can be used in conjunction with antibiotics. Grygorcewicz et al. (2020) in their work presented promising results of the fight against *A. baumannii* biofilm with the use of bacteriophage cocktail combined with antibiotics in a human urine model. The use of a bacteriophage cocktail (Aba-1, Aba-2, Aba-3, Aba-4, and Aba-6) in combination with ciprofloxacin, trimethoprim/sulfamethoxazole, gentamicin, tobramycin, imipenem and meropenem resulted in a significant reduction in the biofilm biomass of *A. baumannii* AB20. The best combination turned out to be the combination of the bacteriophage cocktail with trimethoprim/sulfamethoxazole, where the reduction in biofilm reached 94.3% and 98.6%, with ¼ and ½ MIC, respectively [112].

Studies have also been carried out using the Pyo bacteriophage in the treatment of patients with UTI [97]. Of 118 clinical uropathogens (*P. aeruginosa*, *E. coli*, *Streptococcus* spp., *Proteus mirabilis*, *S. aureus*), 41% of the strains were sensitive to the action of the Pyo bacteriophage, and after its four-fold adaptation, the sensitivity of the bacteria increased to 75%. In the second part of the study, urine samples were collected from nine patients after transurethral resection of the prostate. *E. coli* was detected in the samples of four patients, *Streptococcus* spp. in two, *Enterococcus* spp. in two and *P. aeruginosa* in one of the nine patients. The patients were administered the Pyo bacteriophage intravesically. The bacteriophage was administered twice a day for 7 days (starting from the first day after the treatment) at a volume of 20 mL. The bacteriophage-containing solution was in the bladder for 0.5–1 h. After treatment with bacteriophages, six of nine patients had a 1 to 5 log reduction in urine bacterial counts. Additionally, no side effects of bacteriophage therapy were noted. Other studies show that bacteriophage therapy in treating UTI has not been shown to be more effective than washing the bladder with a placebo solution and standard antibiotic therapy [116].

In addition to the positive aspects, bacteriophage therapy also has limitations. One of them is the possibility of pathogens acquiring bacteriophage resistance. Therefore, bacteriophage cocktails are used due to the lower probability of resistance to all bacteriophages present in the cocktail [101]. In addition, UTI can be caused by more than one bacterial strain, therefore monotherapy in comparison to bacteriophage cocktails may turn out to be unsuccessful [117]. Another limitation of bacteriophage therapy may be the limited access of bacteriophages to biofilm-building bacteria—due to the layers of extracellular polymeric substances (EPS) surrounding the bacteria [118]. A negative effect of bacteriophage therapy may be the adverse response of the immune system due to the sudden lysis of bacteria and the release of toxic lipopolysaccharides [119]. A serious limitation of bacteriophage therapy is that there is not much evidence that the use of bacteriophages alone (without antibiotics) is capable of combating UTIs [120].

Although bacteriophage therapy is not an approved method of treating UTI, further research will optimize this method and determine the role of bacteriophages in the fight against UTI [116]. The results suggest that bacteriophage therapy has great potential in treating UTIs, which needs confirmation in the case of UTI caused by *A. baumannii* strains.

## 2. Conclusions

The species *Acinetobacter baumannii* has been classified to the list of the ten most serious threats to public health and as a critical priority group. Currently, many bacteria, including *A. baumannii* [121], exhibit resistance to a wide spectrum of available antibiotics [122]. Consequently, antibiotics cannot be considered as a real and effective agent against bacterial infections caused by these pathogens. Moreover, in addition to being resistant to many antimicrobial agents, the *Acinetobacter* species has many characteristics that facilitate its spread and increase pathogenicity, such as the aforementioned ability to produce enzymes that allow more efficient acquisition of ingredients necessary for the survival of bacteria or the ability to escape from the human immune system.

An infection of the urinary tract is common and causes many difficulties during treatment due to the antibiotic resistance among bacterial strains and the side effects observed after and/or during antibiotic therapy, therefore other treatments for UTI should be considered [123,124,125,126,127]. Previous studies have shown that UTIs pose serious medical, social and economic problems [6,7,8]. One of the possibilities may be bacteriophage therapy, which can prove to be an effective method to solve this problem. Future studies on the treatment of UTIs caused by uropathogenic strains of *A. baumannii* may have a bearing on the use of this solution in humans in the future. Further research should focus on developing an animal model of UTI caused by *A. baumannii*. Appropriately selected bacteriophages may constitute the composition of a bacteriophage cocktail capable of fighting infections caused by *A. baumannii* [128]. Future studies in animal models may lead to the development of a treatment model for *A. baumannii* UTI in humans.

## Figures and Tables

**Table 1:** Anatomical sites of isolation of *A. baumannii* clinical strains.

Total Isolate Number	Anatomical Site of *A. baumannii* Isolation (%)					Region of the World	Collection Date	Reference
	Urinary Tract	Respiratory Tract	Soft Tissue/Musculoskeletal	Endovascular	Other			
7046	20.4	38.7	26.3	6.2	8.5	Hong Kong	01.1990–11.1994	[47]
1532	28.7	28.3	21.2	12.7	9.1	Spain	1991–1996	[48]
4484	9.9	48.9	13.9	18.3	9.0	USA	1.1994–12.2011	[49]
826	9.0	36.2	4.2	46.0	4.6	Latin America	1.1997–12.2001	[50]
1444	18.0	35.0	18.0	25.0	4.0	USA	1.1998–12.2005	[51]
58	29.3	0.0	63.8	6.9	0.0	Nigeria	2001	[52]
66	6.1	33.3	7.6	53.0	0.0	Canada	2007–2009	[53]
2273	22.2	33.9	31.9	10.4	1.5	USA	1.2007–7.2017	[43]
167	10.2	32.3	44.3	6.0	7.2	Malaysia	10.2010–4.2011	[54]
1176	10.8	26.8	42.3	9.4	10.8	Saudi Arabia	1.2010–12.2013	[55]
140	13.6	0.0	37.1	22.9	26.4	India	8.2010–7.2011	[56]
645	8.8	77.2	5.3	2.0	1.0	Japan	10.2012–3.2013	[57]
100	13.0	59.0	15.0	12.0	1.0	Iran	5.2015–7.2016	[58]
19,957	17.1	39.5	22.7	13.2	7.5	Total		
